# Vision-related quality of life and visual outcomes from cataract surgery in patients with vision-threatening diabetic retinopathy: a prospective observational study

**DOI:** 10.1186/s12955-017-0751-4

**Published:** 2017-09-02

**Authors:** Bijun Zhu, Yingyan Ma, Senlin Lin, Haidong Zou

**Affiliations:** 1 Department of Ophthalmology, Shanghai General Hospital, Shanghai Jiao Tong University School of Medicine, No. 100 Haining Road, Shanghai, 200080 China; 2 Department of Preventative Opthalmology, Shanghai Eye Disease Prevention and Treatment Center, No. 380 Kangding Road, Shanghai, 200040 China

**Keywords:** Cataract, Diabetic retinopathy, Phacoemulsification, Vision-related quality of Life

## Abstract

**Background:**

To examine the benefit of cataract surgery on visual acuity and vision related quality of life in patients with stabilized vision-threatening diabetic retinopathy.

**Methods:**

A total of 126 patients (153 eyes) who were diagnosed with cataract combined with stabilized vision-threatening diabetic retinopathy underwent phacoemulsification. Measurements included the best-corrected visual acuity (BCVA), which was converted into a weighted logarithm of the minimum angle of resolution (logMAR) and vision related quality of life (VRQoL) using the Chinese-version low vision quality of life questionnaire (CLVQOL).

**Results:**

Three months after phacoemulsification, statistically significant improvements were observed in postoperative weighted logMAR BCVA (Z = −9.390 *P* < 0.001). In all of the participants, the CLVQOL total scores (Z = −7.995 *P* <0.001) and four subscale scores including general vision and lighting level (Z = −7.400 *P* <0.001), mobility level (Z = −6.914 *P* <0.001), psychological adjustment level (Z = −8.112 *P* <0.001) and reading, fine work and activities of daily living level (Z = −5.892 *P* <0.001), all improved significantly after the surgeries. Linear regression analyses indicated that the increase in CLVQOL total scores exhibited a significant correlation with the better postoperative weighted logMAR BCVA, greater gain of weighted logMAR BCVA after surgery, bilateral surgery, and longer duration of diabetic retinopathy.

**Conclusions:**

Both visual acuity and the vision related quality of life of the patients with diabetic retinopathy improved significantly after cataract surgery. Cataract surgery is an effective intervention for patients with stabilized diabetic retinopathy.

**Electronic supplementary material:**

The online version of this article (10.1186/s12955-017-0751-4) contains supplementary material, which is available to authorized users.

## Background

Diabetic retinopathy (DR) is a common and specific microvascular complication of diabetes mellitus (DM), and remains the leading cause of preventable blindness in working-aged people [1]. Vision-threatening diabetic retinopathy, which leads to severe visual impairment in diabetic patients, is defined as the presence of severe non-proliferative diabetic retinopathy (NPDR) or proliferative diabetic retinopathy (PDR) and/or diabetic macular edema (DME, also called clinically significant macular edema (CSME)) [2]. Treatments of vision-threatening diabetic retinopathy include laser therapies, anti-vascular endothelial growth factor (VEGF) therapy and vitrectomy [1]. Although the treatments are effective for the preservation of sight in proliferative diabetic retinopathy and macular edema, their effects to reverse visual losses are poor. China is one of the countries with the largest number of people suffering from diabetes mellitus [3]. In 2015, there are more adults with diabetes in China than any other country worldwide, with 109.6 million affected people [4]. The prevalence of diabetic retinopathy was 25–43% among diabetic residents in China [5, 6], and one-third of patients with diabetic retinopathy might develop vision-threatening diabetic retinopathy [1], which accounts for approximately 10 million patients in China and has become a serious public health concern.

Cataract is another common complication of diabetes. There is a high risk of developing cataract in diabetic patients, and the aging factor also contributes to this prevalence. Cataract elicits severe detriments to the quality of life and productivity of these individuals. Although low vision acuity of these patients was caused by both vision-threatening diabetic retinopathy and severe cataract, the severity of cataract is the primary standard for doctors to choose surgery. It is well known that visual acuity can be improved after cataract surgery, but for patients with vision-threatening diabetic retinopathy, both doctors and patients themselves hesitate to choose cataract surgery because there is always minimal improvement of central visual acuity after surgery. However, their visual acuity will worsen and fundus observation will be more difficult if these patients do not elect to have cataract surgery.

It is generally recognised that it is insufficient to evaluate visual function by merely examining central visual acuity. There have been many studies on the topics of vision-related quality of life (VRQoL), which is a subjective observation that differs from central visual acuity. In our previous studies, we found that VRQoL evaluated using the Chinese-version low vision quality of life questionnaire (CLVQOL) was significantly improved in patients with advanced glaucoma or advanced age-related macular degeneration after cataract surgery [7–9], although the central visual acuity improved at low levels. These observations indicate that it is worth performing cataract surgery in these patients. At the present, no study has demonstrated whether the VRQoL improves after cataract surgery in patients with vision-threatening diabetic retinopathy. Thus, we observed the changes in VRQoL before and after cataract surgery in patients with stabilized vision-threatening diabetic retinopathy. This study aimed to provide insight towards the decision-making process of performing cataract surgery in these patients.

## Methods

### Patients and study design

This prospective study enrolled patients who underwent cataract surgery at the Department of Ophthalmology, Shanghai General Hospital, Shanghai Jiao Tong University between January 2014 and June 2015. The inclusion criteria were set as follows: (a) Patients with previous slit-lamp biomicroscopy and fluorescein angiography examinations that confirmed bilateral diabetic retinopathy, who had received treatment including local/grid/panretinal photocoagulation, vitrectomy or intravitreal anti-VEGF injection to control the progress of the disease. The diabetic retinopathy in these patients had not progressed for more than 6 months before the cataract surgery. (b) Patients with the explicit diagnosis of cataract in the operated eye (nuclear hardness grade ≥ 3 (nuclear hardness classification by Emery and Little [10])) were willing to undergo cataract surgery. (c) Patients who could understand and cooperate in this study and were willing to undergo ophthalmic examinations and sign an informed consent. (d) Patients without other eye diseases, such as keratopathy, glaucoma, dacrycystitis, uveitis, ocular trauma, and age-related macular degeneration, etc. (e) Patients who could follow-up within 90 days (3 months) after surgery. Participants were informed of the purpose and the risks of the surgery and that it was uncertain whether the surgery would produce improvement in their visual acuities. All of the patients underwent successful phacoemulsification with foldable posterior chamber intraocular lens implantation through clear corneal incisions under local anaesthesia. There were no severe intra-operative or postoperative complications that occurred. Written informed consents were signed by all of the participants, and the study was approved by the ethics committee of Shanghai General Hospital, Shanghai Jiao Tong University and performed according to the Declaration of Helsinki.

The patients had been monitored for 3 months. The basic information of the patients, including name, gender, age, education level, DM and DR/DME duration, etc., was collected at baseline. Best-corrected visual acuity (BCVA) and VRQoL were the major observation indicators in this study. BCVA (measured at 5 m using a Snellen E chart), and quality of life (measured by CLVQOL) [11] were evaluated 3 days before and 3 months after cataract surgery, respectively. If the patient underwent a second-eye cataract surgery during the 3-month postoperative period, the data, including the BCVA of both eyes and VRQoL, were collected 3 months after the second-eye cataract surgery.

### Measurement of VRQoL

The CLVQOL questionnaire was used in this study, which was translated from the original English-language Low Vision Quality of Life (LVQOL) questionnaire [12], to assess the alteration in vision-related quality of life. The CLVQOL had been applied to measure the quality of life in cataract, glaucoma, AMD, and other ophthalmopathies, with relatively high reliability and validity [7–9, 13, 14]. It included 25 close-ended items, which were all graded on an ordinal scale between 1 (great difficulty due to vision) and 5 (no problem due to vision). These items could also be scored as no longer possible due to vision (attributed a grade of 0) or as not relevant to the patient in their daily lives (attributed the average score of their total responses to avoid the bias in the results of patients who had less items that were relevant compared to others). The CLVQOL total scores ranged from 0 (representing binocular no perception of light) to 125 (representing the best vision function). The 25 items were grouped into 4 subscales: general vision and lighting, (from item 1 to 7); mobility, (from item 8 to 12); psychological adjustment, (from item 13 to 16); and reading, fine work and activities of daily living (from item 17 to 25). The patients were required to complete the questionnaires by themselves. However, if the patients were unable to read or write due to the poor eyesight or lack of education, they were randomly assigned to the investigators (Zou HD or Zhu BJ). The investigators read the questions and recorded the answers chosen by the patients themselves.

### Statistical analysis

The outcomes of our analysis were the changes in BCVA and CLVQOL scores, including subscale scores between the 3-days preoperative and 3-months postoperative study visits. According to the generally-used visual acuity loss classification recommended by the international classification of diseases, ninth revision, clinical modification (ICD-9-CM), the BCVA of the eyes that underwent cataract surgery were classified as <1/20 (blindness or profound visual impairment), ≥1/20 and <1/10 (severe visual impairment), ≥1/10 and <3/10 (moderate visual impairment), and ≥3/10 (normal vision). To compare the visual acuity change in patients after the surgery, the BCVA fractions were all converted into a logarithm of the minimum angle of resolution (logMAR) BCVA [15] and the weighted logMAR BCVA was calculated from the logMAR BCVA of both eyes. (Weighted logMAR BCVA = (0.75 × logMAR acuity in best eye) + (0.25 × logMAR acuity in worst eye)) [16] A logMAR visual acuity of 2.2 was assigned for a finger count, with the lowest visual acuity level in the study. To ensure the internal consistency and reliability of the CLVQOL in the patients, Cronbach’s α coefficients were calculated for the preoperative and postoperative total CLVQOL items. A Cronbach’s α coefficient of more than 0.7 was considered reliable for the measurement. Due to the CLVQOL scores, the preoperative and postoperative weighted logMAR BCVA did not follow a normal distribution (Kolmogorov-Smirnov test, *P*<0.05), and the medians and the corresponding minimum and maximum ranges were calculated. In addition, the Wilcoxon signed rank test was used to compare the differences in these parameters before and after surgery. The tests were two-tailed, and a value of *P*<0.05 was considered statistically significant.

A linear regression analysis was performed to examine the potential factors associated with a change in VRQoL. The changes in CLVQOL total scores and the four subscale scores were defined as the values of CLVQOL scores at 3-months postoperative follow-up minus those before surgery. The independent variables included age, gender, education time (divided into ≤6,7–12, or ≥13 years), duration of DR/DME (divided into ≤5, 6–10, 11–15, or 16–20 years), the performance of bilateral cataract surgery, postoperative logMAR BCVA, and differences between postoperative and preoperative weighted logMAR BCVA. Differences were considered statistically significant at *p* < 0.05. All of the data were analysed using SPSS version 16.0.

## Results

### Descriptive statistics

**Fig. 1:**
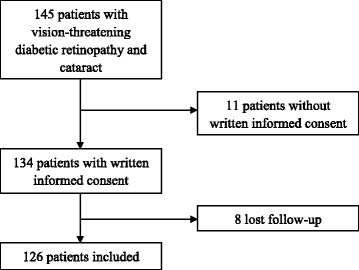
Flowchart of the inclusion and exclusion of the participants in the study

One hundred and forty-five patients were diagnosed as having vision-threatening diabetic retinopathy combining cataract with nuclear hardness of grade 3 or higher and were willing to receive cataract surgery during the study period. Among them, 11 patients did not sign the written informed consent and eight patients were unable to revisit after 3 months (Fig. 1). A total of 126 patients, 62 males and 64 females, 45–82 years old, were involved in the study, including 27 patients (21.4%) with bilateral cataract surgery. The patients’ demographic characteristics are shown in Table 1. A total of 12 patients (9.5%) had missing items in their initial answers, however the maximum number of missing items were 3 (12% of the total items) for an individual patient.

**Table 1:** Baseline characteristics

Age (years, mean ± SD)	65.60 ± 9.23 (range: 45–82)
Gender (male/female)	62/64
Education(0–6 years/7–12 years/≥13 years)	1/34/91
Duration of diabetes (years, mean ± SD)	13.77 ± 9.02
Group of duration of diabetes (years)-- n(%)	
≤ 5	33 (26.2%)
6–10	24 (19.0%)
11–15	15 (11.9%)
16–20	16 (12.7%)
21–25	21 (16.7%)
26–30	17 (13.5%)
Time since diagnosis of DR/DME (years, mean ± SD)	7.26 ± 3.53
Group of time since diagnosis of DR/DME (years)-- n(%)	
≤ 5	39 (31.0%)
6–10	67 (53.2%)
11–15	16 (12.7%)
16–20	4 (3.2%)
Unilateral/bilateral cataract surgery	27/99

### Changes in visual acuity and VRQoL

The changes in visual acuity loss classification for BCVA in the operated eyes are shown in Table 2. The visual acuity reached or exceeded 3/10 (Snellen) in 55 (35.95%) eyes. Compared to the preoperative level, the postoperative BCVA of 60 eyes (39.22%) was not promoted to a higher level. Among these 60 eyes, 49 eyes (81.67%) indicated postoperative BCVA below 3/10 (Snellen).

**Table 2:** Change in BCVA in operated eyes before and after cataract surgery

	Postoperative BCVA			
Preoperative BCVA	<1/20	≥1/20 and <1/10	≥1/10 and <3/10	≥3/10
<1/20	3	8	19	8
≥1/20and < 1/10	0	6	22	4
≥1/10and < 3/10	0	0	40	32
≥3/10	0	0	0	11

*BCVA* best corrected visual acuity, *ICD-9-CM* international classification of diseases, ninth revision, clinical modification

Postoperative weighted logMAR BCVA improvement was achieved in 92.86% of the patients (117/126). Nine patients (7.14%) did not change the weighted logMAR BCVA. The variation of weighted logMAR BCVA was statistically significant after surgery. The weighted logMAR BCVA improved from 0.82 ± 0.34 preoperatively to 0.58 ± 0.30 after 3 months and the total scores of CLVQOL improved from 76.02 ± 24.82 preoperatively to 95.35 ± 20.65 after 3 months (Wilcoxon signed rank test, *P* < 0.001).

**Fig. 2:**
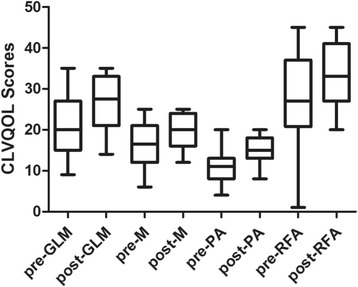
Scores in every subscale of the CLVQOL before and after cataract surgery. GLM: general vision and lighting. M: mobility . PA: psychological adjustment. RFA: reading, fine work and activities of daily living

Cronbach’s α coefficients of the preoperative and postoperative CLVQOL questionnaires were 0.963 and 0.975, respectively. Changes in every subscale of the CLVQOL after cataract surgery are shown in Fig. 2. The CLVQOL total scores and the four subscale scores all improved significantly after surgery (Wilcoxon signed rank test, *P* < 0.001). The median of the change in CLVQOL total scores was 13.5 (range − 47 to 90). There was a 37.5% increase in the median of CLVQOL scores in general vision and lighting level, a 36.4% increase in the psychological adjustment level, a 22.2% increase in reading, fine work and activities of daily living level, and a 21.2% increase in mobility level. There were significant differences in every item of the CLVQOL after surgery (Wilcoxon signed rank test, *P* < 0.001, Additional file 1). In the nine patients whose weighted logMAR BCVA did not improve after cataract surgery, the CLVQOL total scores increased in four patients, remained unchanged in two patients, and decreased in three patients after surgery.

In the linear regression analyses, the total CLVQOL scores improvement was associated with bilateral surgery, better postoperative visual acuity, larger gain of visual acuity, and longer duration of DR, after adjusted for age, gender and education level (Table 3). These factors also correlated with change of scores in the four subscales of the CLVQOL (Table 3).

**Table 3:** Linear regression analyses for change of total scores and the four subscales of the CLVQOL

		GL	M	PA	RFA	Summary
Postoperative logMAR VA	β	−6.5***	−2.0	−9.0***	−10.8***	−28.3***
	95%CI	−10.0, −3.1	−4.4, 0.5	−11.2, −6.8	−16.3, −5.2	−39.0, −17.5
Change of logMAR VA	β	−13.5***	−8.9***	−6.8***	−24.6***	−53.7***
	95%CI	−18.8, −8.1	−12.7, −5.0	−10.3, −3.3	−33.3, −15.9	−70.5, −37.0
Bilateral surgery	β	3.4*	2.6**	1.5	7.4**	14.8***
	95%CI	0.8, 6.0	0.8, 4.4	−0.2, 3.1	3.2, 11.5	6.8, 22.8
Duration of DR (years)						
1–5		Reference	Reference	Reference	Reference	Reference
6–10	β	0.4	0.1	−0.7	0.6	0.3
	95%CI	−1.9, 2.7	−1.6, 1.7	−2.2, 0.8	−3.1, 4.3	−6.8, 7.5
11–15	β	−1.8	−2.0	−2.1	−4.5	−10.3
	95%CI	−5.3, 1.8	−4.6, 0.5	−4.4, 0.2	−10.2, 1.3	−21.4, 0.8
16–20	β	−4.5	−5.5*	−5.2**	−13.9**	−29.1**
	95%CI	−10.5, 1.5	−9.8, −1.2	−9.1, −1.3	−23.6, −4.2	−47.9, −10.4

Adjusted for age, gender and education level

*stands for *P* < 0.05, **stands for *P* < 0.01, and ***stands for *P* < 0.001

*GL* General vision and lighting, *M* Mobility, *PA* psychological adjustment, *RFA* Reading, fine work and activities of daily living

## Discussion

Many authors have reported poor visual outcomes after cataract surgery in patients with diabetic retinopathy, particularly in patients with a history of diabetic retinopathy or diabetic macular edema: Pollack [17] reported that the operated eyes with pre-existing diabetic retinopathy tended to develop clinical cystoid macular edema, and the final visual acuity could be in the range of 6/15–6/30. Schatz [18] found that eyes with diabetic retinopathy with cataract surgery did poorly in terms of visual acuity with no eyes achieving 20/20 or 20/25, only three eyes achieving 20/30 or 20/40, and 16 eyes achieving 20/100 or worse in the study. Only 33% of patients with diabetic macular edema had improved visual acuity as reported by Chiu [19]. They recommended that cataract surgery in patients with diabetic retinopathy be deferred until visual acuity is markedly reduced. Although the visual acuity improved at a low level after cataract surgery in patients with vision-threatening diabetic retinopathy, in our opinion, it should not be the only measurement to determine whether the surgery is necessary. In addition to the traditional objective measurements, such as visual acuity, quality of life subjectively perceived by the patients has been incorporated in more ophthalmic studies. In addition to visual acuity, quality of life outcome assessments provide more information, such as the effect of diseases on psychological function and daily activity [20, 21]. Thus, it is helpful for researchers to evaluate quality of life when they are assessing the effect of diseases and the effect of treatment to provide information for clinical practice. There has been no study on the changes in VRQoL and visual acuity after cataract surgery in patients with stabilized vision-threatening diabetic retinopathy thus far. The current study represents the first report to demonstrate the alterations in VRQoL and visual acuity after cataract surgery in patients with stabilized vision-threatening diabetic retinopathy, with an observed significant improvement in vision-related quality of life.

This prospective study showed that improved vision acuity could be expected in a majority of patients with diabetic retinopathy after phacoemulsification and posterior chamber intraocular lens implantation, albeit at low levels, while the VRQoL improved notably in total scores, in every subscale and for every item of the CLVQOL. In the four subscales of CLVQOL, the postoperative median scores of the general vision and lighting level and the psychological adjustment level increased more. This finding may be due to the obvious increase in scores of the general vision and lighting level and increase in BCVA in most patients after cataract surgery. We propose that it is worthwhile for patients with stabilized vision-threatening diabetic retinopathy to undergo cataract surgery.

Cataract surgery was once considered to increase the risk of the progression of diabetic retinopathy postoperatively [22]. However, recent studies have indicated cataract surgery does not increase the risk of progression of proliferative diabetic retinopathy or macular edema if treated adequately [23]. In our study, all of the participants had received specific treatments to control the progress of the disease before cataract surgery and their diabetic retinopathy did not progress for more than 6 months. This finding indicates that the vision-threatening diabetic retinopathy stabilized. All of the patients remained in their preoperative diabetic retinopathy subsets after the 3-month postoperative follow-up. On the other hand, fundus can be easily observed after cataract surgery. The progress of diabetic retinopathy will be detected as soon as possible. It is beneficial for these patients to have proper treatment of diabetic retinopathy. In this regard, cataract surgery may actually improve long-term outcomes of patient with diabetic retinopathy.

There were some limitations in our study: first, our study focused primarily on the adult Chinese population in Shanghai. Thus, our findings might not be generalisable to the entire population due to racial and cultural differences, which may affect how participants respond to items in the standardised questionnaires. Second, there were no objective measurements of contrast sensitivity, glare sensitivity and visual field that might have captured other components of visual function that were not explained by visual acuity alone [24]. Third, the postoperative follow-up period was relatively short since we performed our evaluation 3 months after the cataract surgery was performed on the patients. Progression of diabetic retinopathy, degenerative retinal neural function and posterior capsular opacification may deteriorate the vision acuity and vision-related quality of life in the long term. Posterior capsular opacification can be cured by Nd:YAG laser, the others were due to both aging and diabetic retinopathy and had no relationship with cataract surgery history.Thus, future studies examining patients of different races with a longer follow-up period and more methods of measurement will further support our findings in this study. In addition, the application of investigators for those who are unable to read or write might cause potential bias, since some patients might report better outcome in face of the investigators, while some patients might complain and report worse outcome than their real situation. Last but not the least, lacking of technique support, rasch model was not used in the present study. Therefore, the results should be interpreted with caution, since the respondent might not fall on a linear scale representing the degree of quality of life change.

## Conclusion

In summary, our data suggested that cataract surgery of phacoemulsification and posterior chamber lens implantation elicited a satisfactory postoperative prognosis for patients with stabilized vision-threatening diabetic retinopathy. Both BCVA andthe VRQoL improved significantly after cataract surgery. Thus, ophthalmologists could consider performing cataract surgery after thorough communications with patients and careful preoperative preparation.

## Additional file

Additional file 1: Changes in every item of CLVQOL after cataract surgery. (DOCX 16 kb)

## Abbreviations

BCVA: Best corrected visual acuity
CLVQOL: Chinese-version low vision quality of life questionnaire
CSME: Clinically significant macular edema
DM: Diabetes mellitus
DME: Diabetic macular edema
DR: Diabetic retinopathy
ICD-9-CM: International classification of diseases, ninth revision, clinical modification
logMAR: Logarithm of the minimum angle of resolution
LVQOL: Low vision quality of life
NPDR: Non-proliferative diabetic retinopathy
PDR: Proliferative diabetic retinopathy
VEGF: Vascular endothelial growth factor
VRQoL: Vision-related quality of life
