# Supplementary material for: Vision-related quality of life and visual outcomes from cataract surgery in patients with vision-threatening diabetic retinopathy: a prospective observational study
Source: Health Qual Life Outcomes. 2017 Sep 2;15:175. doi: 10.1186/s12955-017-0751-4 (PMC5581410; doi:10.1186/s12955-017-0751-4)
Supplement: Additional file 1: — Changes in every item of CLVQOL after cataract surgery. (DOCX 16 kb) [file 12955_2017_751_MOESM1_ESM.docx]

Changes in every item of CLVQOL after cataract surgery

| CLVQOL Item | | Preop CLVQOL Score | | Postop CLVQOL Score | | Wilcoxon signed rank test | | |  |
| --- | --- | --- | --- | --- | --- | --- | --- | --- | --- |
|  |  | Median | Range | Median | Range | | Z Value | *P* value | |
| General vision and lighting | 1. with your eyes getting tired | 3 | 1-5 | 4 | 1-5 | | -6.310 | <0.001 | |
|  | 2. with your vision at night inside the house | 3 | 1-5 | 4 | 1-5 | | -6.318 | <0.001 | |
|  | 3. Getting the right amount of light to be able to see | 3 | 1-5 | 4 | 2-5 | | -6.504 | <0.001 | |
|  | 4. with glare | 3 | 1-5 | 4 | 1-5 | | -5.638 | <0.001 | |
|  | 5. seeing street signs | 3 | 1-5 | 4 | 1-5 | | -5.974 | <0.001 | |
|  | 6. seeing the television | 3 | 0-5 | 3.5 | 0-5 | | -4.598 | <0.001 | |
|  | 7. seeing moving objects | 3 | 1-5 | 4 | 2-5 | | -4.142 | <0.001 | |
| Mobility | 8. with judging the depth or distance of items | 3 | 0-5 | 4 | 2-5 | | -5.004 | <0.001 | |
|  | 9. seeing steps or curbs | 3 | 0-5 | 4 | 2-5 | | -5.614 | <0.001 | |
|  | 10. getting around outdoors | 3 | 0-5 | 4 | 2-5 | | -5.626 | <0.001 | |
|  | 11. crossing a road with traffic | 3 | 1-5 | 4 | 1-5 | | -4.899 | <0.001 | |
|  | 12. with your vision in general | 3 | 1-5 | 4 | 2-5 | | -6.507 | <0.001 | |
| psychological adjustment | 13. unhappy at your situation in life | 3 | 1-5 | 4 | 2-5 | | -6.971 | <0.001 | |
|  | 14. frustrated at not being able to do certain tasks | 3 | 1-5 | 4 | 1-5 | | -7.283 | <0.001 | |
|  | 15. restricted in visiting friends or family | 3 | 1-5 | 4 | 1-5 | | -6.176 | <0.001 | |
|  | 16. how well has your eye condition been explained to you | 3 | 1-5 | 4 | 1-5 | | -6.700 | <0.001 | |
| Reading, fine work and activities of daily living | 17. reading large print | 3 | 0-5 | 4 | 1-5 | | -4.659 | <0.001 | |
|  | 18. reading newspaper text and books | 3 | 0-5 | 4 | 1-5 | | -6.278 | <0.001 | |
|  | 19. reading labels | 3 | 0-5 | 4 | 1-5 | | -5.772 | <0.001 | |
|  | 20. reading your letters and mail | 3 | 0-5 | 4 | 1-5 | | -5.567 | <0.001 | |
|  | 21. having problems using tools | 3 | 0-5 | 4 | 1-5 | | -5.666 | <0.001 | |
|  | 22. finding out the time for yourself | 3 | 1-5 | 4 | 1-5 | | -4.196 | <0.001 | |
|  | 23. Writing | 3 | 0-5 | 4 | 1-5 | | -3.528 | <0.001 | |
|  | 24. reading your own hand writing | 3 | 0-5 | 4 | 1-5 | | -4.108 | <0.001 | |
|  | 25. with your everyday activities | 4 | 0-5 | 4 | 2-5 | | -4.075 | <0.001 | |

CLVQOL = Chinese-version low vision quality of life
